# Telling Your Right Hand from Your Left: The Effects of DNA Supercoil Handedness on the Actions of Type II Topoisomerases

**DOI:** 10.3390/ijms241311199

**Published:** 2023-07-07

**Authors:** Jeffrey Y. Jian, Neil Osheroff

**Affiliations:** 1 Department of Biochemistry, Vanderbilt University School of Medicine, Nashville, TN 37232, USA; jeffrey.y.jian@vanderbilt.edu; 2 Department of Medicine (Hematology/Oncology), Vanderbilt University School of Medicine, Nashville, TN 37232, USA

**Keywords:** DNA topoisomerase, DNA topology, supercoil handedness, type II topoisomerase, DNA relaxation, DNA cleavage

## Abstract

Type II topoisomerases are essential enzymes that modulate the topological state of DNA supercoiling in all living organisms. These enzymes alter DNA topology by performing double-stranded passage reactions on over- or underwound DNA substrates. This strand passage reaction generates a transient covalent enzyme–cleaved DNA structure known as the cleavage complex. Al-though the cleavage complex is a requisite catalytic intermediate, it is also intrinsically dangerous to genomic stability in biological systems. The potential threat of type II topoisomerase function can also vary based on the nature of the supercoiled DNA substrate. During essential processes such as DNA replication and transcription, cleavage complex formation can be inherently more dangerous on overwound versus underwound DNA substrates. As such, it is important to understand the profound effects that DNA topology can have on the cellular functions of type II topoisomerases. This review will provide a broad assessment of how human and bacterial type II topoisomerases recognize and act on their substrates of various topological states.

## 1. Introduction

DNA is often visualized as a ladder. However, the stacking of the nucleotide base pairs upon one another introduces a twist in the structure, converting the ladder into a double helix in which one DNA strand is wrapped around the other [1]. Because of the double-stranded nature of DNA and its extreme compaction into a crowded cellular environment, this plectonemic coiling leads to a number of topological problems in DNA [2,3,4,5].

As long as the ends of DNA are fixed in space, topological properties are defined as those that cannot be changed without breaking one or both strands of the double helix [2,3,4,5,6]. For practical purposes, the ends of cellular DNA can be considered to be fixed in space; they are anchored and unable to rotate freely [3,6]. This is due to the high frictional energy associated with the extreme length of chromosomes in humans, the circular nature of plasmids and chromosomal DNA in bacteria, and the tethering of DNA to chromosomal scaffolds in humans and membranes in bacteria [3,5]. Although the genetic information is organized in a linear array of nucleotide bases, DNA topology plays an important role in facilitating access to this information [2,3,6,7,8].

**Figure 1:**
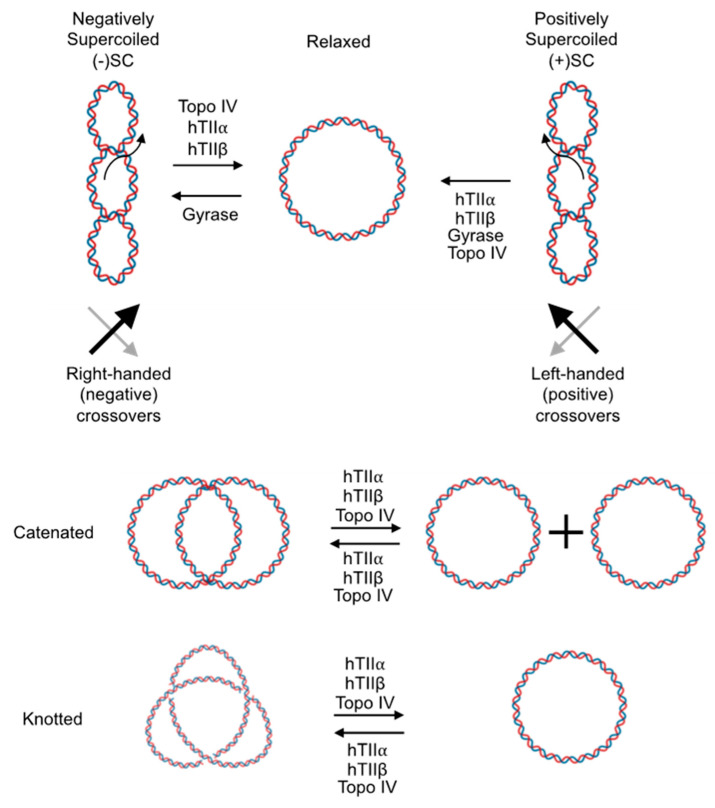
Topological states of DNA. DNA containing no torsional stress is considered “relaxed” (**top middle**). Underwinding or overwinding the DNA results in negatively supercoiled [(−)SC, **top left**] or positively supercoiled [(+)SC, **top right**] DNA. DNA supercoiling is depicted as writhe for visual clarity, but twist and writhe are interconvertible within these molecules. Intermolecular catenanes (**middle**) and intramolecular knots (**bottom**) can also form in DNA. In these cases, twist and writhe are not interconvertible. Type II enzymes (human topoisomerase IIα, hTIIα; human topoisomerase IIβ, hTIIβ; gyrase; topoisomerase IV, topo IV) that can perform each of the reactions to alter topological states are also listed. Created with BioRender.com.

The topology of DNA is described by three concepts: twist, writhe, and linking number [2,3,4,5,9,10]. Twist is the total number of double helical turns in a given DNA segment and represents the torsional stress that is present in the double helix at any time. By convention, positive twist (right-handed twist) is present in the normal right-handed Watson–Crick DNA structure (Figure 1). Writhe is defined as the number of times the double helix crosses itself if the DNA segment is projected in two dimensions and represents axial stress in the molecule. The directionality of the double helical crossover (i.e., node or juxtaposition) is assigned a positive or negative value based on its orientation (i.e., handedness). Positive supercoils [(+)SC] form left-handed crossovers in the double helix, while negative supercoils [(−)SC] form right-handed crossovers.

Linking number represents the sum of twist and writhe. Assuming that the ends of DNA are “fixed” and the double helix is intact (i.e., unbroken), linking number is invariant. DNA duplexes that are not under torsional stress, such as that seen in the Watson–Crick structure, are denoted as “relaxed.” In relaxed molecules, the two strands twist around the helical axis once every ~10.4 base pairs (Figure 1) [3,9,11]. DNA under- or overwinding induces torsional stress in the double helix. If this stress is unconstrained and allowed to freely distribute, it will be partially converted into axial stress (writhe) [2,3,5]. In this case, one portion of the DNA will form a “superhelical turn” around another portion of the molecule. Hence, DNA that is under torsional stress (either under- or overwound) is referred to as being “supercoiled”.

**Figure 2:**
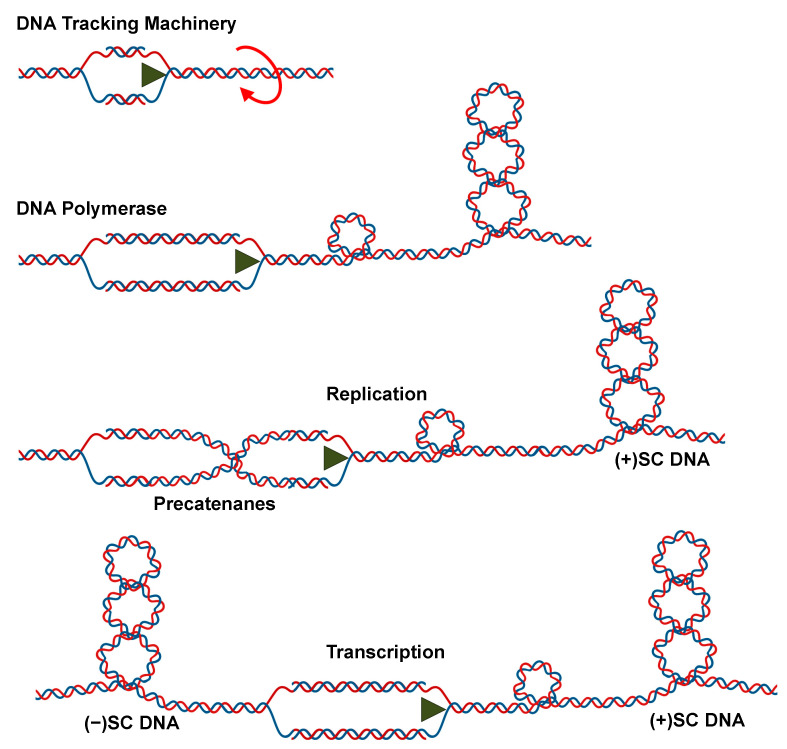
Movement of DNA tracking machinery causes topological problems as indicated by the overwinding shown by the circular red arrow. As DNA tracking systems move through the DNA, twists are pushed ahead of replication forks and transcription complexes, resulting in DNA overwinding and the formation of (+)SCs. In the case of replication, precatenanes also form behind the fork; during transcription, (−)SCs form behind the moving DNA tracking machinery. Created with BioRender.com.

DNA supercoiling is especially relevant to essential nucleic acid processes that require strand separation, such as replication and transcription [2,3,5,12,13,14]. In species ranging from bacteria to humans, the double helix is globally underwound (i.e., negatively supercoiled) ~6% [2,3,5]. This underwound nature of the genome reduces the energy required to separate complementary base pairs, facilitating the opening of the double helix to access the genetic information [15,16,17,18]. In contrast, once the movement of DNA tracking machinery begins, the deleterious effects of DNA topology manifest (Figure 2). Because helicases separate but do not unwind the two strands of DNA, they do not remove any turns of the double helix. Consequently, acutely overwound (i.e., positively supercoiled) DNA forms ahead of the tracking machinery, generating an increase in torsional stress that needs to be alleviated [19]. If unresolved, the accumulation of (+)SCs blocks replication and transcription, causing these processes to stall rapidly [17,18,20,21,22].

Additional topological structures, such as tangles and knots, result from replication and recombination, respectively [5,6,7]. Tangles (i.e., catenanes) can form between distal segments of the same DNA molecule or separate molecules during processes such as replication [2,3,5]. Catenanes must be removed to allow for the proper separation of sister chromatids during mitosis [23,24,25,26,27,28]. Due to the long length of DNA, nucleic acid knots can form within a single DNA molecule during processes such as recombination. DNA knots prevent the separation of the two strands of DNA [2,3,5,7,13].

## 2. Topoisomerases

In order to maintain appropriate levels of DNA supercoiling and remove knots and tangles from the genome, cells encode enzymes known as topoisomerases [3,4,5,6,29,30]. These enzymes are ubiquitous to all domains of life and are necessary for cellular survival. All topoisomerases modulate the topological state of the genome through the creation of transient breaks in the DNA sugar–phosphate backbone. Broadly, there are two classes of topoisomerases, and they are both defined by the number of DNA strands they cleave per enzyme reaction cycle [2,3,5,6,30]. Type I topoisomerases generate a single-stranded break, or “nick”, in the double helix [3,5,30]. In contrast, type II topoisomerases create a double-stranded break in the genetic material [2,3,5,6,29,30,31]. This review will focus on type II topoisomerases.

## 3. Type II Topoisomerases

There are two subclasses of type II topoisomerases: type IIA and type IIB. To date, functional type IIB enzymes have only been identified in plants and archaea and will not be discussed further [30,32,33].

The first type IIA enzyme, bacterial DNA gyrase, was discovered in 1976 [34]. Bacterial topoisomerase IV was later identified in 1990 [35]. Most bacterial species encode gyrase and topoisomerase IV [30,36]. However, a few species, such as *Mycobacterium tuberculosis*, encode only a single type II topoisomerase, gyrase, which can presumably perform the cellular functions of both type II enzymes [4,6,29,37,38].

The first eukaryotic type II enzyme was identified in *Drosophila* in 1980 [39]. *Drosophila* and other invertebrates, as well as lower eukaryotes, such as yeast, encode only one type II enzyme, topoisomerase II. In contrast, vertebrates, such as humans, express two forms of the type II enzyme: topoisomerase IIα and topoisomerase IIβ [3,5,6,7,30,31,40]. Human topoisomerase IIα and topoisomerase IIβ were identified in 1988 [41] and 1989 [42,43], respectively.

## 4. Type II Topoisomerase Domain Structures

**Figure 3:**
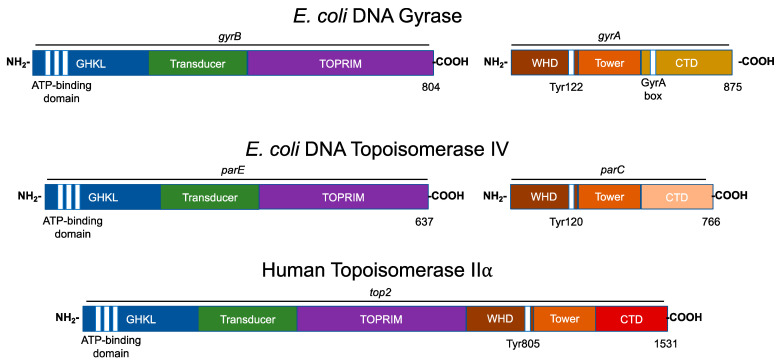
Domain structures of type II topoisomerases. The domain structures of three type II topoisomerases, bacterial (*Escherichia coli*) gyrase and topoisomerase IV, and human topoisomerase IIα are shown. Regions of homology among the enzymes are indicated by colors. The N-terminal (i.e., GyrB) homology domains contain the regions responsible for ATP binding and hydrolysis (GHKL, blue). The vertical white stripes represent the three conserved motifs that define the ATP-binding domain. The N-terminal domain also contains the binding site for divalent metal ions (TOPRIM, purple). The central (i.e., GyrA) region (WHD, brown) contains the active site tyrosyl residue that forms the covalent bond with DNA during scission. For bacterial gyrase, the variable C-terminal domain (gyrase, gold; topoisomerase IV, pink) contains the “GyrA box” that is necessary for the wrapping mechanism. For human topoisomerase IIα, the C-terminal homology domain (CTD, red) contains nuclear localization sequences (NLS) and phosphorylation sites (PO_4_). The active site tyrosine residue is indicated for each enzyme.

Bacterial type II topoisomerases are heterotetrameric in structure (A_2_B_2_; Figure 3). The founding type II enzyme, gyrase, is comprised of two distinct subunits: GyrA and GyrB. Like gyrase, topoisomerase IV is a heterotetramer that is composed of two separate subunits: ParC and ParE (which are homologous to GyrA and GyrB, respectively) in Gram-negative species and the corresponding GrlA and GrlB subunits in Gram-positive species (Figure 3) [35,44].

Eukaryotic type II topoisomerases are homologous to the bacterial type II enzymes [3,5,6,29,30,45]. However, the two bacterial subunits have fused into a single polypeptide in the eukaryotic type II topoisomerases (Figure 3) [3,5,6,29,30,45].

All known type II topoisomerases share several common structural features across three regions. Using DNA gyrase as the model, the N-terminus is located in GyrB, the catalytic core spans portions of GyrB and GyrA, and the C-terminus is located in GyrA (Figure 3) [3,5,6,29,30,45].

The N-terminal region contains the N-gate, where the DNA enters the enzyme. This portion of the molecule includes the ATPase active site, also known as the GHKL (DNA gyrase, Hsp90, bacterial CheA-family histidine kinases, and MutL; Figure 3, blue) domain. The GHKL domain contains an ATP-binding region that is formed from an eight-stranded antiparallel beta sheet surrounded by alpha helices [46,47]. The N-terminal region also contains the transducer domain (Figure 3, green), which relays ATP binding/hydrolysis information to the catalytic core [48,49]. The binding of ATP induces the dimerization of the N-terminal region, which shifts the N-gate into a closed conformation. The bound ATP interacts with a lysine residue in the transducer domain and subsequently facilitates rotation between the GHKL and transducer domains [32,47].

The catalytic core contains the topoisomerase/primase (TOPRIM; Figure 3, purple) domain, which coordinates the active site divalent cations, the winged-helix domain (WHD; Figure 3, brown), which contains the active site tyrosine residue, and the tower domain (Figure 3; orange), which maintains polar and electrostatic interactions with the DNA substrate [48,50,51].

The TOPRIM domain is necessary for the transesterification reaction between the scissile phosphate of the DNA backbone and active site tyrosine residue [47,50]. The active site divalent cation is held by an aspartate-any residue-aspartate (DxD) motif and a glutamate residue that can act as a general acid–base moiety [52,53]. The DxD motif and its coordinate divalent cation in the TOPRIM domain, along with the active site tyrosine of the WHD, enable the formation of the two transient cuts of the DNA backbone via a non-canonical two-metal ion mechanism [6,51,54,55].

The WHD is able to bind DNA and also contains the active site tyrosine residue, which is responsible for the nucleophilic attack on the scissile phosphate of the DNA double helical backbone and the formation of the transient topoisomerase–DNA covalent bond [45,48].

The tower domain functions in DNA bending. This domain contains a beta sheet that can interact with one of the captured DNA double helices (the gate or G-segment, to be discussed later), bending the DNA segment to promote cleavage [56,57,58]. The presence of a conserved, invariant isoleucine residue has been found to intercalate between two base pairs of the G-segment, inducing a ~150° bend [56,59].The deletion or mutation of this isoleucine interferes with proper DNA bending, the subsequent cleavage, and the relaxation of supercoiled DNA [56,59].

The sequence of the C-terminal domain varies considerably between species, but it is characterized by the presence of charged amino acid residues [30,60]. In gyrase, this region contains a seven-amino acid motif known as the GyrA box (Figure 3, gold) [29,36,47,61,62]. The GyrA box is located within a six-blade beta pinwheel in the C-terminal domain, and it uniquely allows for the wrapping of the DNA substrate to introduce (−)SCs [29,36,47,61,62,63].

In comparison to gyrase, the C-terminal domain of topoisomerase IV does not contain the structure necessary to wrap and supercoil DNA (Figure 3, pink). Rather, topoisomerase IV contains a “broken” five- (not six) blade beta pinwheel and lacks a GyrA box [46,64,65,66]. Remnants of the canonical GyrA motif have been found in each of its pinwheel “blades” [64,65]. Nonetheless, the C-terminal domain of topoisomerase IV contains positively charged moieties on its outer surface, suggesting a role in binding DNA [46].

The C-termini of eukaryotic type II topoisomerases also contain the remnants of highly charged pinwheel blades but are inherently disordered in the absence of DNA (Figure 3, red) [67,68]. This portion of the eukaryotic enzyme also contains nuclear localization sequences and sites for posttranslational modifications such as phosphorylation and SUMOylation [5,45,47]. For the IIα isoform, these modifications enable the enzyme to be concentrated at centromeres during mitosis [67,69].

## 5. Catalytic Cycle of Type II Topoisomerases

**Figure 4:**
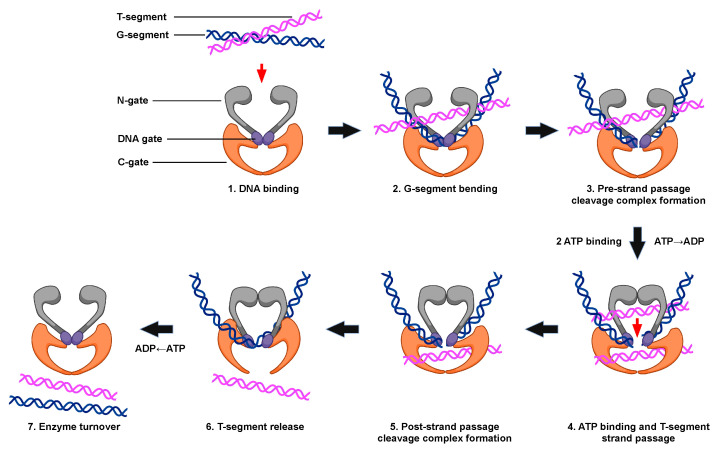
The catalytic cycle of type II topoisomerases. The double-stranded DNA passage reaction of type II topoisomerases can be separated into discrete steps. (1) Type II enzyme binding to two intact segments of DNA: the gate, or G-segment (blue, the first segment bound) and transport, or T-segment (purple, the second segment bound). (2) Bending of the G-segment to assess for sites of DNA cleavage. (3) Double-stranded DNA cleavage of the G-segment (i.e., formation of the pre-strand cleavage complex). (4) Binding of two ATP molecules, which triggers the closing of the N-gate, opening of the DNA gate, and the passage of the T-segment through the DNA gate. Strand passage occurs more rapidly if one of the two ATP molecules is hydrolyzed. (5) Formation of the post-strand passage cleavage complex. (6) Religation of the cleaved G-segment and release of the T-segment through the C-gate of the protein. (7) ATP hydrolysis, which triggers enzyme turnover and the regeneration of the enzyme to initiate a new round of catalysis. Created with BioRender.com.

All type II topoisomerases undergo similar catalytic cycles. These enzymes function by forming a transient double-stranded DNA break and modulate the topological state of DNA by a double-stranded passage reaction (Figure 4) [3,5,6,29,30,31,47]. The enzyme begins its catalytic cycle by capturing a segment of intact DNA through the opened N-terminal region (N-gate, gray) of the enzyme (Step 1). This first segment will be cut by the enzyme and is known as the “gate” or G-segment. The segment that is captured second and eventually transported through the transiently cleaved G-segment is known as the “transport” or T-segment. In the presence of two divalent cations, such as Mg^2+^, and in coordination with the TOPRIM domain, the G-segment is assessed for bendability (Step 2) [57]. DNA sequences that can be bent are distorted to an angle of ~150° and can be used as the site for scission [56,57,58,59].

Both strands of the bent G-segment are then cleaved via a nucleophilic attack by the two active site tyrosine residues on the phosphate backbone of the double helix (Step 3). DNA cleavage is initiated when a general base, which is believed to be a conserved histidine residue, deprotonates the hydroxyl group of the active site tyrosine, allowing the oxyanion to attack the scissile phosphate. Two divalent cation molecules, such as magnesium (i.e., Mg^2+^), are necessary for this nucleophilic attack [5,6,29,30,70]. Type II topoisomerases use a non-canonical two-metal ion mechanism [51,70]. The presence of one divalent cation enables interaction with the bridging 5′-oxygen molecule of the scissile bond and speeds up rates of enzyme-mediated cleavage at the first cut site. Once the first DNA strand is cut, the second strand is cleaved ~20-fold faster [71]. The resulting enzyme-cleaved DNA complex is a transient structure that has the enzyme covalently bound to the scissile 5′-phosphate of the double helical backbone.

To maintain the bond energy of the sugar–phosphate backbone as well as genomic integrity during the double-stranded DNA cleavage process, the type II enzyme forms covalent bonds between the two active site tyrosine residues and the newly generated 5′-phosphate groups of the DNA backbone, generating a phosphotyrosyl linkage and a four-base DNA overhang [5,6,29,31,47]. The transiently cleaved, covalently linked enzyme–DNA structure that is formed is known as the pre-strand passage “cleavage complex” [5,6,29,31]. The formation of the cleavage complex during enzyme catalysis is tightly regulated to prevent the generation of permanent DNA breaks or the disruption of genomic integrity [5,6,29,30].

When ATP enters the enzyme–DNA complex is not precisely known. This high-energy cofactor is not required for either DNA cleavage or religation. However, upon the binding of two ATP molecules, the N-gate is closed, triggering a conformational change in the enzyme that translocates the T-segment through the transient opening in the DNA (i.e., DNA “gate”, Step 4). Although hydrolysis of the high-energy cofactor is not necessary for this strand passage event to occur, this step proceeds faster if one of the two bound ATP molecules is hydrolyzed [72].

After strand passage, a second, post-strand passage, cleavage complex is formed (Step 5). The type II enzyme then religates the cleaved DNA to regenerate the intact DNA double helix. DNA religation is initiated when a general acid removes the hydrogen from the 3′-terminal hydroxyl group [6,48]. Another nucleophilic attack is then initiated on the phosphotyrosyl bond, regenerating the intact DNA double helical backbone and the enzyme active site. The T-segment is then released from the protein (Step 6). The hydrolysis of a second ATP molecule occurs, resetting the type II enzyme conformation and allowing for enzyme turnover during the next cycle of catalysis (Step 7).

## 6. Cellular Functions of Gyrase and Topoisomerase IV

The main function of gyrase is to maintain the proper superhelical density of the bacterial genome (acting in conjunction with the ω protein, a type IA topoisomerase) and to remove (+)SCs that accumulate ahead of DNA tracking machinery (i.e., polymerases and helicases) during essential nucleic acid processes such as replication and transcription [5,7,29,30,36,73,74,75,76]. Because of its ability to wrap DNA during catalysis (discussed below), gyrase functions primarily to generate (−)SCs, which also allows it to remove (+)SCs in a highly efficient manner [63,76,77,78,79].

Because topoisomerase IV is unable to wrap DNA, it functions as a canonical type II topoisomerase [36,63,76,79,80,81]. As such, the enzyme primarily acts to resolve precatenanes formed between daughter chromosomes during DNA replication and remove DNA knots that form during recombination [64,81]. Topoisomerase IV may also play a role ahead of DNA tracking systems, but the precise nature of this process is poorly understood [81,82].

## 7. Cellular Functions of Human Type II Topoisomerases

As discussed earlier, humans encode two isoforms of topoisomerase II: α and β. Topoisomerase IIα and topoisomerase IIβ are distinct in their expression patterns [3,6,29,30,41,42,43,83,84,85]. The levels of topoisomerase IIα are at their lowest during the G_1_ phase and rise throughout the S phase, eventually peaking at the G_2_/M phase boundary [84,85,86]. The enzyme is found almost exclusively in actively proliferating tissues, localizes predominantly in the nucleus, is associated with replication forks and transcription machinery, and is tightly bound to chromosomes and sister chromatids throughout mitosis [28,67,85,86,87,88,89,90]. Topoisomerase IIα is required for the survival of proliferating cells and is believed to be the main isoform that functions in growth-related processes such as replication and chromosomal segregation [4,30,31,47,83,84,88,91,92]. One of the primary functions of the enzyme is to resolve precatenanes that form behind replication forks [4,31,47,83,84,88,91,92]. However, genomic evidence also suggests a role for topoisomerase IIα during transcription [93,94,95].

In contrast to the α isoform, topoisomerase IIβ is not required for survival at the cellular level [40,88,91,96]. The concentration of topoisomerase IIβ is independent of the stage of the cell cycle, and the levels of the isoform are generally consistent irrespective of cell proliferation status [40,47,97]. Although cells can survive in the absence of topoisomerase IIβ, the enzyme is required for proper neural development in mice [31,40,88,98]. It also plays a role in the transcription of hormonally regulated genes [31,40,98,99].

## 8. When Good Enzymes Go Bad

Because type II topoisomerases generate transient double-stranded DNA breaks as requisite intermediates during their catalytic cycles, these enzymes also have the capacity to fragment the genome [6,17,31,100]. Thus, the type II enzymes are dualistic in nature; although essential for survival, they pose an eminent danger to the cell every time they act [6,17,31,100]. Consequently, the equilibrium between the forward cleavage reaction (enabling the subsequent strand passage) and the reverse religation reaction (resealing the DNA break) heavily favors religation to maintain genomic integrity during catalysis. As a result, under normal equilibrium conditions, covalent enzyme-cleaved DNA complexes generated by type II topoisomerases are tightly regulated [3,5,6,17,31,100]. These complexes are present at low steady-state levels, short-lived, and tolerated by the cell [3,5,6,17,31,100]. Cleavage complexes become more lethal when they are formed ahead of DNA tracking systems, such as replication forks and transcription complexes. When polymerases or helicases attempt to traverse the covalently bound topoisomerase “roadblock” in the genetic material, cleavage complexes can become disrupted, leaving the enzyme unable to religate the double-stranded DNA breaks [3,5,6,17,31,100,101]. In these cases, the “non-ligatable” DNA breaks must be repaired by DNA damage response and recombination pathways [3,5,6,17,31,100,101]. These actions can trigger unwanted chromosomal insertions, deletions, translocations, and cell death pathways [6,31,100,101]. Because DNA found ahead of tracking systems is usually overwound, cleavage complexes formed with (+)SC DNA are potentially the most lethal to cells [5,31,100,101,102].

## 9. The Effects of DNA Superhelicity on the Actions of Type II Topoisomerases

Type II topoisomerases play critical roles in a variety of essential nucleic acid processes. Because these enzymes work on negatively and positively supercoiled DNA substrates and removal of these supercoils results in the formation of relaxed DNA, it is important to understand how these enzymes distinguish their substrates and products and how supercoil handedness affects their actions.

### 9.1. Recognition of DNA Substrate versus Product

The earliest experiments on DNA topology recognition by type II topoisomerases focused on the abilities of these enzymes to distinguish their nucleic acid substrates from products. These studies found that type II enzymes interacted more tightly with their DNA substrates. Gyrase was found to bind relaxed DNA (substrate) ~10-fold more tightly than (−)SC DNA (product) [103,104]. For the canonical type II topoisomerases, topoisomerase IV also binds (−)SC (substrate) ~5-fold over relaxed DNA (product) [105]. Both yeast and *Drosophila* topoisomerase II can sense DNA supercoiling, preferentially binding with (−)SC over relaxed DNA [106,107]. *Drosophila* topoisomerase II also hydrolyzes ATP more rapidly in the presence of underwound DNA substrates [108]. Later, it was found that human topoisomerase IIα displayed higher affinities for supercoiled over relaxed DNA substrates [108,109]. Finally, human topoisomerase IIα maintains higher levels of cleavage complexes with (−)SC over relaxed DNA molecules, although the sites of cleavage remain the same [110].

It has been proposed that canonical type II topoisomerases distinguish supercoiled molecules from relaxed molecules by recognizing the presence or absence of DNA crossovers (i.e., writhe) during binding. Electron microscopy studies of *Drosophila* topoisomerase II-DNA complexes have shown that the enzymes strongly prefer to bind at sites of DNA juxtaposition independent of torsional stress [111]. A later study demonstrated that topoisomerase II simultaneously bound two double-stranded DNA segments and that the binding activity was independent of catalytic activity [112].

### 9.2. Recognition of Supercoil Handedness during DNA Strand Passage

#### 9.2.1. DNA Relaxation and Supercoiling

Eventually, the field of type II topoisomerases transitioned away from distinguishing supercoiled DNA from relaxed DNA and toward the recognition of supercoil handedness. The global underwinding (negative supercoiling) of the genome puts energy into the DNA and enables the separation of the double helix, whereas DNA overwinding (positive supercoiling) ahead of tracking systems has the capacity to impede essential nucleic acid processes. Consequently, it is critical to understand how type II topoisomerases distinguish supercoil handedness during catalysis.

Early works on *Drosophila* and yeast topoisomerase II found that both enzymes were unable to distinguish (−)SC or (+)SC DNA during catalytic reactions and that they relaxed both substrates at comparable rates [107,109,113,114].

Similar to the type II topoisomerases from lower eukaryotes, human topoisomerase IIβ removes (−)SCs and (+)SCs at similar rates [109,115]. However, a major distinguishing characteristic between human topoisomerase IIα and topoisomerase IIβ is that the α isoform can discern supercoil handedness during strand passage and relaxes (+)SC 10–fold faster than it does (−)SC DNA [109,115]. Several lines of evidence indicate that this difference between topoisomerase IIα and topoisomerase IIβ results from elements in their respective C-terminal domains [115,116,117]. First, the C-terminal domain is the most varied region of type II topoisomerases. For example, the C-terminal domains of human topoisomerase IIα and topoisomerase IIβ display only ~31% sequence similarly, whereas the other regions of the enzymes display ~79% sequence similarity [30,37,40]. Second, the deletion of the C-terminal domain of topoisomerase IIα abrogates the ability of the enzyme to preferentially relax (+)SC substrates [109,115]. Even the deletion of a single “pinwheel blade” from the C-terminal domain of the enzyme decreases its ability to distinguish supercoil handedness [115]. Third, in experiments that switched the C-terminal domains of topoisomerase IIα and topoisomerase IIβ, a topoisomerase IIα enzyme that carried the C-terminal domain of the IIβ isoform lost the ability to distinguish supercoil handedness, whereas a chimeric topoisomerase IIβ enzyme that carried the C-terminal domain of the α isoform gained the ability to preferentially relax (+)SC DNA [109,115].

The ability of topoisomerase IIα to preferentially remove (+)SCs is due to a recognition of writhe, rather than twist, in its DNA substrate [118]. Presumably, the path that (+)SC DNA follows on the enzyme interacts with the C-terminal domain in a manner (not yet understood) that enhances the rate of strand passage.

Later studies examined the ability of the bacterial type II topoisomerases to distinguish supercoil geometry during strand passage. Gyrase removes (+)SC DNA at least 10-fold faster than it introduces (−)SCs into relaxed substrates [82,119,120]. This rapid removal of positive supercoils requires the GyrA box in the C-terminal domain of gyrase, which enables the DNA wrapping mechanism of the enzyme [82]. However, DNA wrapping cannot completely explain the recognition of supercoil handedness by gyrase, as mutant enzymes that lack this feature can still relax (+)SC DNA ~two-fold faster than (−)SC substrates [note that because wild-type gyrase normally underwinds DNA, it is not able to relax (−)SCs] [82,119,120,121].

Although topoisomerase IV primarily works behind replication forks as a decatenase, it also preferentially removes (+)SC versus (−)SC DNA [82,119]. Similar to findings with human topoisomerase IIα, the loss of the C-terminal domain impedes the ability of *E. coli* topoisomerase IV to distinguish DNA supercoil geometry during strand passage [64,66]. The ability of topoisomerase IV to distinguish supercoil handedness during DNA strand passage also appears to be based on writhe [118,122].

#### 9.2.2. DNA Catenation/Decatenation

While DNA relaxation is performed via intramolecular strand passage of supercoiled substrates, intermolecular strand passage is necessary to resolve catenanes (i.e., tangles). Type II topoisomerases can also recognize topology during the catenation/decatenation reaction. Yeast topoisomerase II is capable of sensing supercoil handedness during the catenation/decatenation reaction [113]. The enzyme preferentially decatenates (−)SC over (+)SC DNA but favors catenating (+)SC over (−)SC DNA [113]. Similar to results with DNA relaxation, human topoisomerase IIα is able to distinguish between different supercoiled states of DNA during catenation, whereas topoisomerase IIβ cannot. Unexpectedly, the α isoform catenates underwound molecules faster than it does overwound substrates, which is antithetical to the preference during relaxation reactions (human topoisomerase IIα relaxes overwound substrates faster) [123]. In contrast to results with human topoisomerase IIα, topoisomerase IV preferentially catenates (+)SC DNA, which parallels results with relaxation experiments [123].

### 9.3. Recognition of Supercoil Handedness during DNA Cleavage

Type II topoisomerases can also recognize supercoil geometry during DNA cleavage [63,82,109,117,119,124,125]. However, this recognition differs from that which occurs during the DNA strand passage reaction. For example, even though topoisomerase IIα is the only human type II enzyme that can distinguish DNA topology during strand passage, both topoisomerase IIα and IIβ maintain two- to four-fold higher levels of cleavage complexes on (−)SC versus (+)SC DNA [109,115,117]. Furthermore, the ability to recognize supercoil handedness during DNA cleavage lies within the catalytic core of the enzymes as opposed to the C-terminal domain [110]. Consequently, type II topoisomerases appear to recognize supercoil handedness in a bimodal manner, using different mechanisms to distinguish DNA geometry during different catalytic events.

Similar to the human type II enzymes, gyrase also maintains two- to four-fold higher levels of cleavage complexes with (−)SC over (+)SC DNA. However, gyrase (at least the enzyme from *M. tuberculosis*) requires elements in the N-terminal domain to enable the recognition of supercoil handedness during DNA cleavage [119]. Because cleavage complexes formed on (+)SC DNA are the most dangerous, the fact that the human type II enzymes and gyrase generate lower levels of cleavage on overwound substrates make them safer for the cell.

In contrast to the above enzymes, topoisomerase IV shows no large difference in levels of cleavage generated with (−)SC versus (+)SC DNA [82,119,126,127]. However, because topoisomerase IV appears to work behind replication forks, this lack of discrimination during DNA cleavage may have less impact on the cell [82].

It is notable that the recognition of DNA topology during cleavage is not altered by the presence of anticancer or antibacterial drugs; human topoisomerase IIα, topoisomerase IIβ, and gyrase maintain higher levels of cleavage complexes with (−)SC over (+)SC substrates, whereas topoisomerase IV maintains similar levels [128].

The differential recognition of supercoil handedness by type II topoisomerases during DNA cleavage cannot be explained by rates of religation of the cleaved DNA, either in the absence or presence of drugs [128]. Topoisomerase IIα and gyrase form more stable cleavage complexes with (−)SC DNA in the presence of anticancer and antibacterial drugs, respectively [128]. However, in the absence of drugs, the lifetimes of cleavage complexes for all the human and bacterial type II topoisomerases are short and do not vary due to supercoil handedness [128]. Thus, while the stability of cleavage complexes may, under specific circumstances, contribute to the recognition of supercoil handedness, it cannot fully explain how the type II enzymes distinguish DNA geometry during cleavage [128]. Rather, the abilities of the human type II topoisomerases and bacterial gyrase to discern supercoil handedness during scission appear to reflect the forward rates of DNA cleavage. These enzymes all cleave (−)SC DNA faster than they do (+)SC substrates [128]. In contrast, topoisomerase IV, which does not discriminate supercoil handedness during DNA cleavage, cleaves underwound and overwound substrates at similar rates [128]. Again, the above relationships hold in the absence of anticancer or antibacterial drugs [128].

It is not known whether type II topoisomerases utilize twist or writhe to recognize supercoil handedness during DNA cleavage. However, because the portions of the enzymes involved in this recognition are so limited compared to those required during strand passage, it is not clear how DNA writhe could contribute to the recognition of supercoil geometry during cleavage. An intriguing possibility is that this recognition is dependent on DNA twist. To this point, the twist of underwound DNA aligns with the angle of gate opening during the double-stranded DNA passage reaction [129], whereas the twist in overwound DNA should oppose gate opening. Further studies are necessary to better understand the roles of twist and writhe in the recognition of supercoil geometry during DNA cleavage.

## 10. Conclusions

The globally underwound state of DNA in cells and the formation of overwound nucleic acid structures ahead of replication forks, transcription complexes, and other DNA tracking systems have important ramifications for proper biological function. To perform their critical cellular roles, type II topoisomerases transiently cut both strands of the DNA, open the double helix, and pass another nucleic acid segment through the DNA gate. Similar to other enzymes, the type II topoisomerases can distinguish their substrates from their products. In most cases, these enzymes can also distinguish between different DNA substrates. Type II topoisomerases that have been implicated in functioning on overwound DNA during replication or transcription often remove (+)SCs faster than (−)SCs and maintain lower levels of cleavage complexes with overwound substrates. These properties make these enzymes safer for the cell. Conversely, those that act primarily behind replication forks do not share these abilities. Thus, the ability of type II topoisomerases to recognize DNA supercoil geometry appears to have adapted to their unique cellular functions.

## Data Availability

Data sharing not applicable.
